# Dietary flavonoids, lignans and colorectal cancer prognosis

**DOI:** 10.1038/srep14148

**Published:** 2015-09-15

**Authors:** Raul Zamora-Ros, Elisabeth Guinó, M. Henar Alonso, Carmen Vidal, Mercè Barenys, Antonio Soriano, Victor Moreno

**Affiliations:** 1 Biomarkers Group, Nutrition and Metabolism Section, International Agency for Research on Cancer (IARC), Lyon, France; 2 Cancer Prevention and Control Program, Catalan Institute of Oncology (ICO); 3 Biomedical Research Institute of Bellvitge (IDIBELL); 4 CIBER en Epidemiologia y Salud Pública (CIBERESP), Barcelona, Spain; 5 Gastroenterology Service, Hospital de Viladecans, Barcelona, Spain; 6 Gastroenterology Service, University Hospital Bellvitge (HUB); 7 Department of Clinical Sciences, Faculty of Medicine, University of Barcelona (UB), Barcelona, Spain

## Abstract

Flavonoids and lignans are polyphenol classes with anticarcinogenic activities against colorectal cancer (CRC). However, very limited epidemiological evidence exists on their effects on CRC prognosis. This study aimed to evaluate the association between flavonoid and lignan intakes with the risk of CRC recurrence and overall survival in CRC patients. The study followed incident histologically confirmed CRC cases in Barcelona (Spain). Validated dietary questionnaires and lifestyle information were collected at recruitment. An ad hoc food composition database on flavonoids and lignans was compiled by using data from the US Department of Agriculture and Phenol-Explorer databases. Adjusted hazards ratios (HR) and 95% confidence intervals (CIs) were estimated using multivariable Cox models. After 8.6 years of mean follow-up, 133 of 409 (32.5%) participants died and 77 of 319 (24.1%) had a CRC recurrence. Total flavonoids were associated neither with CRC recurrence (HR comparing extreme tertiles 1.13, 95% CI 0.64–2.02; P-trend 0.67) nor with overall survival (HR_T3vsT1_ 1.06, 95% CI 0.69–1.65; P-trend 0.78) in the multivariable models. No associations were also observed with either total lignans or any flavonoid subclass intake. In conclusion, the results of the current study do not support a role of flavonoid and lignan intake in the CRC prognosis.

Flavonoids and lignans are classes of polyphenols found in plant-based foods, such as fruits, vegetables, tea, wine, chocolate and cereal products^1^. They have been largely investigated due to their anticarcinogenic properties including their ability to induce differentiation and apoptosis, and inhibit metabolic carcinogen activation, cell proliferation, tumor cell adhesion and invasion, and angiogenesis^2^,^3^. Flavonoid and lignan intakes have been inversely associated with colorectal cancer (CRC) risk in several case-control studies, but not in cohort studies^1^,^4^,^5^,^6^. Only one epidemiological study has evaluated the potential effect of flavonoids with respect to CRC prognosis^7^. The Polyp Prevention Trial suggested that high intakes of flavonols and isoflavones, two flavonoid subclasses, were related to a decreased risk in advanced colorectal adenoma recurrence^7^. To the best of our knowledge, no studies has been performed to evaluate the association between lignan intake and CRC prognosis.

In the current study, we prospectively investigated the association between the intake of total flavonoids, lignans and flavonoid subclasses, and the risk of colorectal recurrence and overall survival in the Belvitge Colorectal Cancer Study.

## Methods

### Subjects and study design and ethics

Incident histologically confirmed CRC cases were recruited at the University Hospital of Bellvitge, Barcelona, Spain, from January 1996 to December 1998, as described elsewhere^8^. A total of 523 histologically confirmed CRC cases were identified, of whom 424 participated in the study (81% participation rate). For this analysis of prognosis, 15 patients were excluded because died within 60 days of surgery due to complications or chemotherapy toxicity, thus 409 will be analyzed. The study was performed in accordance with relevant guidelines and regulations. All participants gave written consent and the Ethics Committee of the Bellvitge University Hospital approved the study protocol.

### Dietary and lifestyle assessment

Data collection included a validated dietary history enquiring about usual intakes in the previous 12 months, anthropometric data, and questionnaires on sociodemographic factors, lifestyles and health history. Intakes of dietary flavonoid and lignan aglycones were estimated as described previously^8^ using the US Department Agriculture^9^, Phenol-Explorer^10^ and the United Kingdom Food Standards Agency^11^ databases. Furthermore, our database was expanded using retention factors, recipes, and estimations based on similar food or food group items and logical zeros. The final database contained 1,877 food items and only 10% of missing values.

### Outcome assessment

Health outcome data was collected through active follow-up. Oncologists updated electronic clinical records at the stablished follow-up visits, recording recurrence events. Mortality events were extracted from the hospital administrative database, with biannually updates. The end points of interest were overall survival, which included death related to CRC, and CRC recurrence. The analysis of recurrence was restricted to patients with R0 resection (without residual tumor after surgery) and excluded patients with metastasis at diagnosis that could not be resected (stage IV), thus only 319 patients were considered. Patients with no events of interest were censored at date of last information (up to November 2014). More than 75% of the patients had at least 5 years of follow-up.

### Statistical analyses

Cox proportional hazards models were used to estimate hazards ratios (HRs) with 95% confidence intervals (CI) for the association between flavonoid and lignan intakes and CRC recurrence risk and overall survival. Proportional hazards assumption was assessed using a test of the interaction of effects with time. Also graphs based on Schoenfeld residuals were used, which showed no evidence of deviation. The intakes of total and subclasses of flavonoids and lignans were analyzed as tertiles using both absolute amounts adjusted for energy and as density variables (mg/1000 kcal*d), with similar results (data not shown). Multivariable models were adjusted for sex, age (tertiles), total energy (tertiles) and CRC stage (I, II, III, IV). BMI, smoking status, physical activity, intake of alcohol, fruits, vegetables, red and processed meat were not included in final models because they did not change effect estimates >10%. We calculated P-value in the risk categories analysis by the trend test. Interactions with sex, age, BMI, tobacco consumption and physical activity were examined but were not significant. Data were analyzed with the statistical software R version 3.1.2 (R Foundation for Statistical Computing, Vienna, Austria).

### Power calculation

Using Power (version 3.0, 1999, National Cancer Institute, Bethesda, MD), we calculated that, given our size and α = 0.05, our study had 80% power to detect an HR ≥ 2.0.

## Results

After 8.6 years of mean follow-up, 133 of 409 (32.5%) participants died and 77 of 319 (24.1%) had a CRC recurrence. The median (interquartile range) intake of total flavonoids and lignans were 196.4 (119.8–308.4) mg/d and 0.7 (0.5–1.0) mg/d, respectively. Flavanols were the most important contributor (76.0%, including flavan-3-ol monomers and proanthocyanidins) to total flavonoid intake, followed by flavanones (9.2%), flavonols (7.6%), anthocyanidins (6.0%), flavones (1.1%), and isoflavones (0.1%). The richest food sources of total flavonoids were fruits (65.1%), wine (14.4%), legumes (6.3%) and vegetables (4.4%). For lignans, the main contributors were fruits (32.3%), vegetables (31.2%), and cereal products (10.7%). Baseline characteristics of the participants are shown in Table 1.

Total flavonoid intake was not associated with either CRC recurrence risk ((hazard ratio comparing third to first tertile (HR_T3–T1_) 0.97, 95% CI: 0.60–1.56; P trend = 0.89) or overall survival (HR_T3–T1_ 0.87, 95% CI: 0.47–1.62; P trend = 0.71) in the multivariable models (Table 2). The results obtained for total lignan or flavonoid subclasses did not show any association either.

## Discussion

The present prospective study shows no association between the intake of total flavonoid, flavonoid subclasses, and the CRC recurrence. Similar results were observed in the Polyp Prevention Trial between any colorectal adenoma recurrence and total flavonoid and flavonoid subclass intake^7^. However, they suggested inverse associations between flavonol and isoflavone intakes and advanced adenoma recurrence^7^. These findings may not be generalizable to other populations, as it was well discussed by Bobe *et al.*, because most of the participants were healthy conscious^7^. Moreover, the flavonol intake was greater in the trial than in the standard United States population^12^, although it was much lower than the European intakes^13^. Despite of that, in the Bellvitge colorectal cancer case-control study, we did not find any association between flavonol and isoflavone intake and CRC risk^8^. No association between total lignan intake and CRC recurrence was found in the current study, which is the first one evaluating this topic.

To the best of our knowledge, this is the first study evaluating the association between flavonoid and lignan intakes and the survival of CRC patients. In the present study, no association was observed with the intake of both total flavonoids and lignans, and any flavonoid subclass. Similar results have been shown in the Spanish EPIC study, where the cancer mortality was not related to the intake of flavonoids and lignans^14^. The main strength of the current study is the prospective design, with active long follow-up of recurrence events. Inherent to prospective studies, we had some lost of follow-up, but more than 75% of the patients had at least 5 years of information. Probably the major limitations of the study are related to the potential measurement error in the dietary intake assessment, which may have produced an underestimation of the effects. However, dietary questionnaires were previously validated for some flavonoid-rich foods, such as fruits, vegetables, tea, and wine^15^. Moreover, our database was expanded with the most comprehensive databases on flavonoids and lignans^9^,^10^,^11^. Another limitation is the potential modification of diet related to cancer diagnosis or during the follow-up of the study, which cannot be considered because only diet prior to cancer diagnosis was assessed by the questionnaire.

In summary, our results do not support a role of flavonoid and lignan intake in the CRC prognosis, suggesting that they are not associated with an improvement in both the overall survival and CRC recurrences in CRC patients.

## Additional Information

**How to cite this article**: Zamora-Ros, R. *et al.* Dietary flavonoids, lignans and colorectal cancer prognosis. *Sci. Rep.* **5**, 14148; doi: 10.1038/srep14148 (2015).

## Figures and Tables

**Table 1:** Baseline characteristics of the subjects in the Bellvitge colorectal cancer study by recurrence and vital status.

**Characteristic**	**No recurrence**	**CRC recurrence**	**Alive**	**Deceased**	
n	242	77	276	133	
Men, n (%)	144 (59.5%)	45 (58.4%)	159 (57.6%)	89 (66.9%)	
Median age (25–75^th^), years	67.5 (59.0–74.0)	67.0 (56.0–74.0)	67.0 (59.0–74.0)	68.0 (58.0–75.0)	
Median BMI (25–75^th^), kg/m^2^	26.1 (23.4–29.3)	25.0 (23.0–28.1)	26.0 (23.4–29.1)	25.0 (23.1–27.2)	
Current smokers, n (%)	37 (15.3%)	11 (14.3%)	44 (15.9%)	28 (21.1%)	
High physically active, n (%)	126 (52.3%)	41 (53.2%)	137 (49.8%)	75 (56.4%)	
CRC type, n (%)					
Colon	150 (62.0%)	43 (55.8%)	168 (60.9%)	87 (65.9%)	
Rectum	92 (38.0%)	34 (44.2%)	108 (39.1%)	45 (34.1%)	
CRC stage, n (%)					
I	61 (25.2%)	4 (5.2%)	64 (23.1%)	4 (3%)	
II	93 (38.4%)	29 (37.7%)	103 (37.4%)	22 (16.6%)	
III	88 (36.4%)	46 (57.2%)	102 (37%)	47 (35.3%)	
IV			7 (2.5%)	60 (45.1%)	
Median total energy (25–75^th^), kcal/d	2027 (1614–2610)	1967 (1698–2587)	2003 (1605–2574)	2026 (1640–2583)	
Median alcohol (25–75^th^), g/d	0.3 (0–18.0)	0 (0–9.8)	0.3 (0–16.8)	0 (0–16.1)	
Median vegetables (25–75^th^), g/d	157.3 (80.9–253.2)	159.2 (109.8–279.4)	158.3 (83.7–264.1)	155.8 (85.2-279.4)	
Median fruit (25–75^th^), g/d	230.3 (146.8–337.1)	212.7 (147.7–319.8)	230.0 (147.5–338.8)	214.8 (122.5–347.8)	
Median red meat (25–75^th^), g/d	38.4 (19.9–64.1)	43.4 (22.7–63.9)	38.8 (21.4–62.7)	43.4 (18.6–66.6)	
Median processed meat (25–75^th^), g/d	24.6 (10.4–44.9)	27.9 (14.3–59.1)	25.4 (10.7–46.0)	28.6 (14.3–53.6)	
Median flavonoids (25–75^th^), mg/d	189.2 (117.9–323.2)	213.3 (113.8–294.7)	187.7 (119.1–318.6)	219.9 (123.2–302.1)	
Median lignans (25–75^th^), mg/d	0.7 (0.5–1.0)	0.7 (0.5–0.9)	0.7 (0.5–1.0)	0.7 (0.5–0.9)	

Abbreviations: CRC: colorectal cancer; BMI: body mass index.

**Table 2:** Adjusted hazard ratios (HR) and 95% confidence intervals (CI) of the colorectal cancer recurrence and overall survival according to the intake of tertiles of total flavonoid, lignans and flavonoid subclasses in the Bellvitge colorectal cancer study.

	**Overall survival (N = 409)**				**Colorectal cancer recurrence (N = 319)**			
	**Cutoff (mg/d)**	**Deaths**	**HR (95% CI)**	**P-trend**	**Cutoff (mg/d)**	**Cases**	**HR (95% CI)**	**P-trend**
Total flavonoids								
T1	<147.1	43	1 (ref)		<144.5	25	1 (ref)	
T2	147.1–260.3	50	1.00 (0.65–1.53)		144.5–265.3	30	1.22 (0.71–2.10)	
T3	>260.3	40	0.97 (0.60–1.56)	0.89	>265.3	22	0.87 (0.47–1.62)	0.71
Flavanols								
T1	<102.4	42	1 (ref)		<102.4	22	1 (ref)	
T2	102.4–198.8	48	1.02 (0.66–1.58)		102.4–201.7	28	1.33 (0.75–2.34)	
T3	>198.8	43	0.99 (0.63–1.58)	0.98	>201.7	27	1.34 (0.73–2.45)	0.34
Flavan–3-ol monomers								
T1	<10.6	43	1 (ref)		<10.1	27	1 (ref)	
T2	10.6–21.0	45	0.95 (0.61–1.47)		10.1–21.0	26	0.93 (0.53–1.66)	
T3	>21.0	45	0.93 (0.59–1.46)	0.75	>21.0	24	0.91 (0.49–1.67)	0.76
Proanthocyanidins								
T1	<91.7	40	1 (ref)		<89.1	22	1 (ref)	
T2	91.7–177.6	47	1.02 (0.66–1.59)		89.1–179.5	28	1.28 (0.73–2.27)	
T3	>177.6	46	1.08 (0.68–1.71)	0.75	>179.5	27	1.3 (0.71–2.39)	0.39
Flavonols								
T1	<11.0	46	1 (ref)		<11.0	25	1 (ref)	
T2	11.0–18.6	40	0.72 (0.46–1.12)		11.0–18.6	24	0.95 (0.54–1.67)	
T3	>18.56	47	0.90 (0.58–1.39)	0.69	>18.6	28	1.18 (0.65–2.13)	0.60
Flavanones								
T1	<11.5	46	1 (ref)		<11.3	30	1 (ref)	
T2	11.5–25.9	43	1.01 (0.66–1.55)		11.3–25.0	24	0.78 (0.45–1.33)	
T3	>25.9	44	0.92 (0.60–1.42)	0.71	>25.0	23	0.80 (0.46–1.39)	0.40
Anthocyanidins								
T1	<7.2	46	1 (ref)		<7.1	26	1 (ref)	
T2	7.2–17.9	37	0.84 (0.54–1.32)		7.1–17.2	29	1.24 (0.73–2.12)	
T3	>17.9	50	0.91 (0.58–1.44)	0.70	>17.2	22	0.87 (0.48–1.57)	0.68
Flavones								
T1	<1.3	44	1 (ref)		<1.2	27	1 (ref)	
T2	1.3–3.2	42	0.70 (0.45–1.08)		1.2–3.1	26	0.95 (0.55–1.63)	
T3	>3.2	47	0.87 (0.56–1.36)	0.56	>3.1	24	0.97 (0.54–1.73)	0.90
Isoflavones								
T1	<0.2	49	1 (ref)		<0.2	30	1 (ref)	
T2	0.2–0.3	43	0.69 (0.46–1.06)		0.2–0.3	26	0.79 (0.46–1.35)	
T3	>0.3	41	0.97 (0.62–1.53)	0.76	>0.3	21	0.60 (0.33–1.09)	0.09
Lignans								
T1	<0.6	40	1 (ref)		<0.6	28	1 (ref)	
T2	0.6–0.9	52	0.89 (0.56–1.40)		0.6–0.9	27	0.99 (0.57–1.73)	
T3	>0.9	41	0.83 (0.50–1.37)	0.47	>0.9	22	0.68 (0.36–1.26)	0.22

Cox models adjusted for sex, age, total energy and colorectal cancer stage.
